# Large-deformation and high-strength amorphous porous carbon nanospheres

**DOI:** 10.1038/srep24187

**Published:** 2016-04-13

**Authors:** Weizhu Yang, Shimin Mao, Jia Yang, Tao Shang, Hongguang Song, James Mabon, Wacek Swiech, John R. Vance, Zhufeng Yue, Shen J. Dillon, Hangxun Xu, Baoxing Xu

**Affiliations:** 1 Department of Mechanical and Aerospace Engineering, University of Virginia, Charlottesville, VA 22904, USA; 2 Departments of Materials Science and Engineering, University of Illinois at Urbana-Champaign, Urbana, IL 61801, USA; 3 CAS Key Laboratory of Soft Matter Chemistry, Department of Polymer Science and Engineering, University of Science and Technology of China, Hefei, Anhui 230026, China; 4 Department of Engineering Mechanics, Northwestern Polytechnical University, Xi’an, Shaanxi 710072, China; 5 Institute for Nanoscale and Quantum Scientific and Technological Advanced Research, University of Virginia, Charlottesville, VA 22904, USA

## Abstract

Carbon is one of the most important materials extensively used in industry and our daily life. Crystalline carbon materials such as carbon nanotubes and graphene possess ultrahigh strength and toughness. In contrast, amorphous carbon is known to be very brittle and can sustain little compressive deformation. Inspired by biological shells and honeycomb-like cellular structures in nature, we introduce a class of hybrid structural designs and demonstrate that amorphous porous carbon nanospheres with a thin outer shell can simultaneously achieve high strength and sustain large deformation. The amorphous carbon nanospheres were synthesized via a low-cost, scalable and structure-controllable ultrasonic spray pyrolysis approach using energetic carbon precursors. *In situ* compression experiments on individual nanospheres show that the amorphous carbon nanospheres with an optimized structure can sustain beyond 50% compressive strain. Both experiments and finite element analyses reveal that the buckling deformation of the outer spherical shell dominates the improvement of strength while the collapse of inner nanoscale pores driven by twisting, rotation, buckling and bending of pore walls contributes to the large deformation.

Amorphous carbon has been widely acknowledged as one of the most ubiquitous materials in nature. Unlike crystalline carbons such as graphene, carbon nanotube, and diamond which completely consist of either *sp*^2^ or *sp*^3^ hybridized carbon bonds, amorphous carbon is a covalent random network composed of both *sp*^3^ and *sp*^2^ hybridized carbons without grain boundaries^1^,^2^. Such hybridized bonding structures lead to its distinct mechanical properties with high hardness yet sustain little compressive deformation^3^,^4^,^5^. In order to achieve a high compressive deformation and delay brittle failure, several different strategies have been proposed such as doping a small amount of silicon or adjusting the fraction of *sp*^2^ hybridized carbon in the random covalent network^6^,^7^. Other than altering the intrinsic bonding structures at the atomistic scale, another effective approach to improve the mechanical properties is to create porous structures. In contrast to their solid nonporous counterpart, porous carbon is more favorable of sustaining a large deformation by releasing the degrees of deformation freedom through appropriately designed porous structures^8^,^9^,^10^,^11^,^12^,^13^. However, like most porous materials, the introduction of porous structures will dramatically decrease the deformation strength of corresponding materials. Additional structure adjustment is required without sacrificing the strength of porous materials after generation of pores in carbon materials. In fact, many natural materials like nacre and scallop with curviplanar countours and overall spherical geometries have proved to be the best configuration for enhancing mechanical strength^14^,^15^,^16^,^17^. Therefore, creating a hybrid structure down to the nanoscale by taking advantage of porous and shell structures is expected to offer a straightforward route to simultaneously achieve both high strength and large deformation.

Here we report that amorphous carbon nanospheres consisting of an outer spherical shell with porous interior structure can improve strength and compressive deformation simultaneously. The thin outer shell is expected to improve the failure strength, and the interior hierarchical nanopores inherit the advantages of porous materials with large deformation. Meanwhile, the nanosphere represents a zero-dimensional structure and provides an ideal foundation for isotropic mechanical response. Amorphous carbon nanospheres with different inner pore sizes and outer geometric diameters were obtained through pyrolysis of molecular carbon precursors confined inside isolated droplets. *In situ* nanomechanical compression experiments and finite element simulations on individual nanospheres were carried out to elucidate the underlying deformation mechanism responsible for such unique mechanical response, and in turn to offer quantitative guidelines for design and fabrication of future structural carbon materials.

## Results

Amorphous carbon spheres were obtained through a low-cost, simple and controllable ultrasonic spray pyrolysis approach using alkali propiolates (HC≡CCOOM, M = Li, Na, K) as carbon precursors (Supplementary Figure S1). Pyrolysis of bulk precursors only generates fragile amorphous carbon forms, while aerosolized droplets act as isolated microreactors which confine the thermal decomposition of carbon precursors and lead to the formation of porous carbon spheres^18^,^19^. Alkali propiolates containing different cations decompose at different temperatures and produce different amounts of gases, which leads to different thermal decomposition pathways, and allows us to be able to prepare carbon spheres in the submicron regimes with different porous structures (The decomposition process of alkali propiolates can be described as^18^:


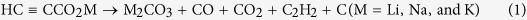


**Figure 1:**
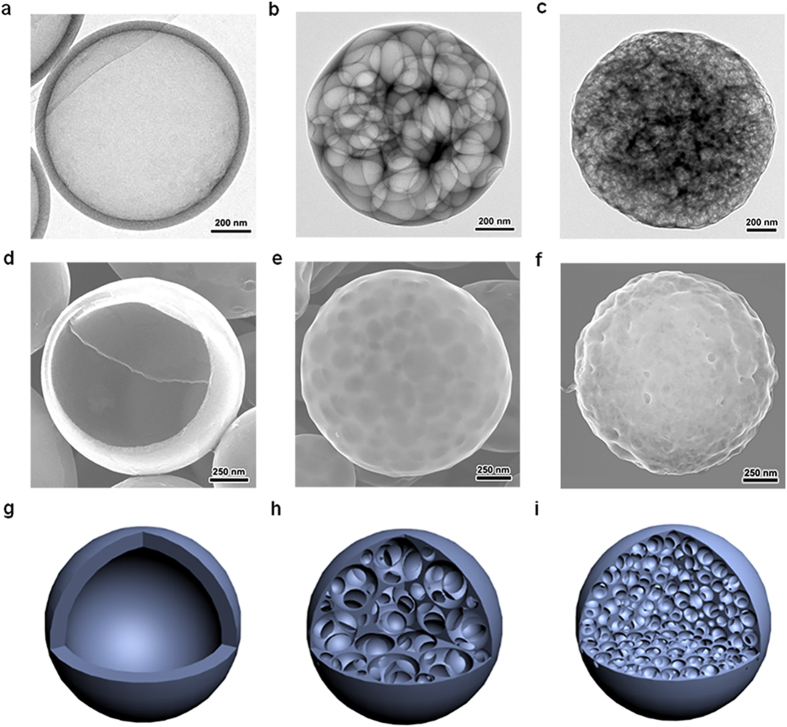
Carbon nanospheres with different pore structures. (**a–c**) TEM images and (**d–f**) SEM images of carbon spheres studied in this work, and their corresponding schematic illustrations (**g–i**).

Figure 1 shows the TEM and SEM images of synthesized porous spheres with different interior pore sizes and their corresponding schematic illustrations. Carbon nanospheres containing macropores (100 ~ 200 nm) separated by thin carbon sheets can be obtained from pyrolyzing potassium propiolates while carbon nanospheres with mesopores (10 ~ 20 nm) can be obtained using lithium propiolates as precursors (Supplementary Figure S2). The thickness of outer shell in macroporous and mesoporous carbon nanospheres is 15 ~ 30 nm and 4 ~ 10 nm, respectively. Moreover, hollow carbon nanospheres were generated using a mixture of lithium, sodium, and potassium propiolates as precursors. The porous carbon spheres synthesized in this approach are amorphous even after heat treatment at 800 °C for 12 hours (Supplementary Figure S3). Raman analysis reveals that the carbon spheres prepared in the above described method consist of both *sp*^2^ and *sp*^3^ hybridized carbon bonds. And there is no dramatic variance of graphitic and aliphatic carbons between different carbon nanospheres as the I_D_/I_G_ ratios of carbon nanospheres with different structures are very close even after heat treatment (Supplementary Figure S3). The density of carbon nanospheres with macroporous and mesoporous pores were 0.21 and 0.26 g/cm^3^ respectively. The density of hollow carbon nanospheres was measured to be 0.47 g/cm^3^. Therefore, the density of porous carbon nanospheres obtained in this approach is approximately one order of magnitude lower than that of solid amorphous carbon (2.0 ~ 2.4 g/cm^3^)^20^,^21^.

**Figure 2:**
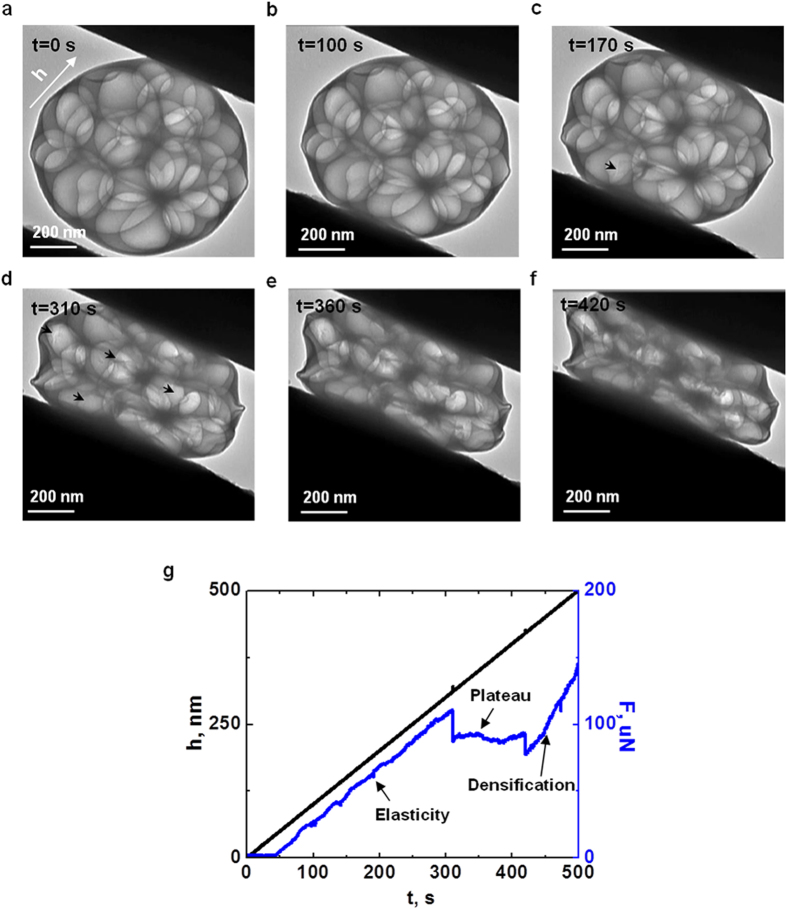
*In situ* compression experiment on a single porous carbon sphere with macroporous structure. (**a–f**) TEM images of a single carbon sphere under the deformation with different loading times (insert white arrow indicates the loading direction). The first crack occurred at t = 170 s and propagated to a sudden failure of the whole carbon sphere with the appearance of more cracks and collapse of pores at 310 s (black arrows point out the position of main cracks). Most pores inside the carbon sphere failed and densified at t = 420 s. (**g**) Applied displacement loading and resulting force curves with compression time concurring with the above deformation process.

*In situ* uniaxial compression tests were performed on individual carbon nanospheres using the Hysitron PI-95 Picoindenter system in TEM (see Experimental Section and Supplementary Figure S4). A single carbon nanosphere was well positioned at the center of a silicon wedge substrate without obvious necking at contact (Supplementary Figure S5), and was compressed at a rate of 1 nm/s via a diamond flat punch with a 3 μm diameter. A macroporous carbon nanosphere 950 nm in diameter (*D*) was first compressed under a monotonic load (Supplementary Video 1). Figure 2a shows the initial position of the nanosphere on the silicon wedge without compressive loading. When the nanosphere was compressed uniaxially by *h* = 60 nm (t = 100 s), the external spherical shell buckled and inner pores deformed elastically without obvious fracture (Fig. 2b). Beyond a critical displacement loading (*h* = 130 nm, t = 170 s), a crack appeared in one of the weak inner pores (Fig. 2c). The external spherical shell did not show any damage, and is expected to experience a post-buckling deformation which is delayed by inner pores. With increasing compression (*h* = 270 nm, t = 310 s), the crack propagated and more inner pores collapsed (Fig. 2d). Almost all inner pores collapsed as the compressive strain increased further (*h* = 320 nm, t = 360 s) (Fig. 2e), while the external shell remained intact but deformed to an elliptical geometry. Finally, all inner pores completely collapsed and the densification phenomena associated with the formation of a solid carbon phase were observed in the final stage of compression (Fig. 2f). The concurrent force and displacement curve along with the above compression process is shown in Fig. 2g which consists of elastic, plateau, and densification regimes, analogous with that of typical porous materials^22^,^23^,^24^. The approximately linear increase under small deformation (*h* < 270 nm, t < 310 s) indicates an elastic deformation on the entire carbon sphere, and the small perturbations are caused by discrete bending and buckling deformation events of the external spherical shell and inner pores. A compressive force plateau is observed followed by a force drop beyond the compression time of 310 s (*h* = 270 nm). As observed in deformation process shown in Supplementary Video 1, this plateau is associated with the bending, buckling, twisting, rotation, and collapse of the majority of pores. The small undulations of amplitude in force plateau represent the nonuniformity of randomly generated pores in carbon nanospheres. In addition, no expansion (even inward folds) vertical to displacement loading direction occurs, indicating nearly zero Poisson’s ratio which further implies the deformation mechanism commenced by bending, buckling and collapsing of inner localized porous structures. These commensal deformation mechanisms indicate an excellent inheritance from the deformation behaviors of biological structures of shells and honeycombs^15^,^25^,^26^,^27^, and facilitate the enhancement of both strength and deformation in hybrid pore-shell structures.

**Figure 3:**
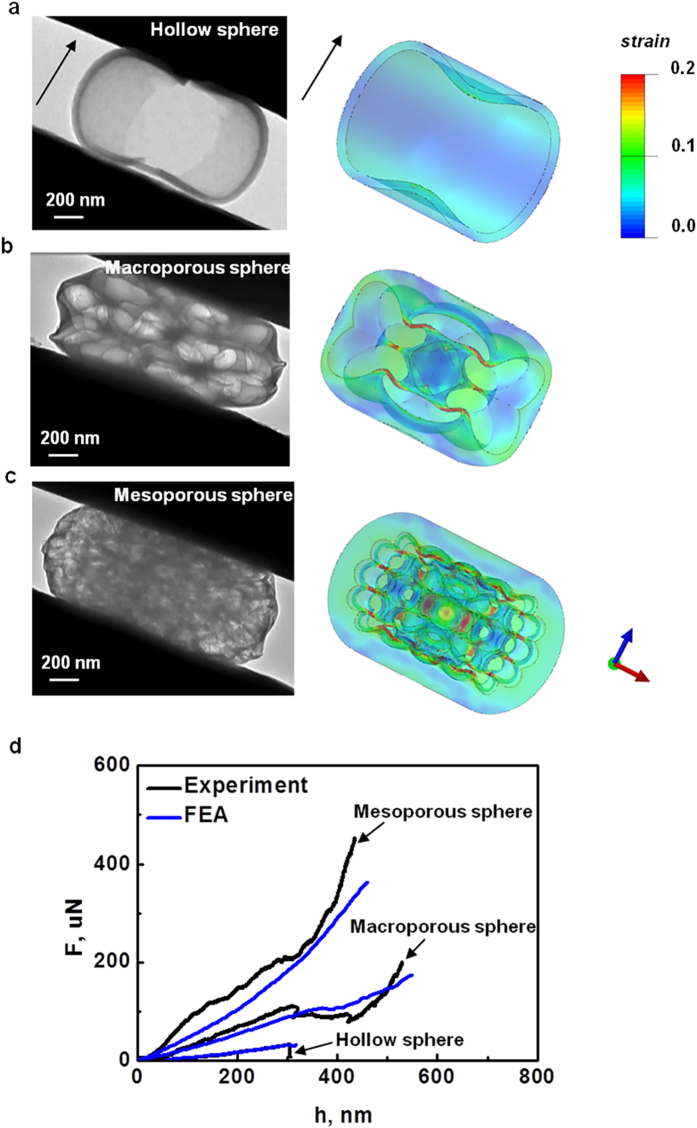
Mechanical responses of amorphous carbon nanospheres with different structures under the same compressive strain. Experiment and FEA results of (**a**) hollow, (**b**) macroporous, and (**c**) mesoporous carbon nanospheres at the same compressive strain (28%, *h* = 270 nm, arrow represents the loading direction). An obvious failure is observed in hollow carbon nanosphere while not in loose and mesoporous carbon nanospheres. (**d**) Variation of compressive force with displacement loading. Comparisons between experiment and FEA show a good agreement in key features.

To investigate the mechanical behavior of this novel spherical porous structure composed of amorphous carbon, we further performed the compression experiments on carbon spheres with different pore sizes (Fig. 3). At the compressive strain (defined as the ratio of compressive displacement to spherical diameter) of 28% (*h* = 270 nm), a failure of the entire hollow carbon sphere is observed (Fig. 3a, Supplementary Video 2). At the same compressive strain, the carbon sphere with macropores (Fig. 3b, Supplementary Video 1) still deforms elastically due to the “deformation resistance” of inner pores which significantly delays the failure of the entire sphere. In addition, the mesoporous carbon sphere does not fail at the same compressive strain (28%, *h* = 270 nm) either (Fig. 3c, Supplementary Video 3). The improvement in force plateau is expected to be caused by the hierarchical porous structure, where partial densification to solid occurs once small nanopores collapse. Consequently, the plateau duration becomes unconspicuous and extends to the densification regime early.

Finite element analysis (FEA) has been carried out to provide quantitative understanding on the deformation of the spherical shell and localized inner pores. The intrinsic Young’s modulus of solid carbon, *E*_*s*_, is first extracted from a compression experiment performed on a solid carbon nanosphere using Hertz contact theory (Supplementary Figures S6 and S7, Supplementary Note 1). The measured value is 20.2 GPa and is used in all FEA. An excellent consistence between FE simulations and experiments on the hollow carbon nanosphere is shown in Fig. 3a (Supplementary Figures S8 and S9, Supplementary Video 4). These hollow carbon spheres deform elastically upon compression until fracture events occur with a sudden drop of compressive force. Compared with the inherent strength-driven brittle failure of solid carbon spheres, the bending and buckling of outer spherical shell delay the fracture to large deformations. Similar delayed failure by bucking and bending mechanism has also been observed in hollow cadmium sulphide^28^ and silica^29^ spheres.

In order to quantitatively understand the deformation mechanism of carbon spheres with different numbers of pores, 21 and 125 pores with different sizes were symmetrically positioned inside a 950 nm carbon nanosphere in FEA to represent the macro- and mesoporous structures in experiments, respectively (FE simulation method, Supplementary Note 2, Supplementary Figure S8). Both force and displacement curves simulated from FEA show similar features to those from compression tests. Note that a quantitative matching is difficult given the complexity of pore density, shapes, distribution, and even the thickness of outer shell (Supplementary Figure S10), and the simulation only shows a qualitative match with experimental results but all the key features are captured. Particularly, the collapse plateau of pores is well predicted in a carbon sphere containing 21 pores. Increasing the relative density of pores by adjusting pore numbers (Supplementary Figures S11) or pore sizes (Supplementary Figures S12) raises the plateau force and reduces the plateau duration beyond which densification starts. Furthermore, unlike the deformation process of hollow carbon spheres, the outer spherical shell buckles first in porous carbon spheres and then adjacent pores bend, buckle, collapse and propagate toward the center of the sphere (Supplementary Videos 5 and 6). The deformed carbon spheres at the compressive strain of 28% (*h* = 270 nm) based on FEA are given in Fig. 3. Obvious buckling of pores in the macroporous carbon sphere is observed, while bending deformation is dominated in the mesoporous carbon sphere. More importantly, as indicated in simulated deformation process (Supplementary Videos 5 and 6), the inner pores will rotate and twist in the mesoporous carbon nanosphere before failure occurs, thus increasing both plateau force and duration. The rotation and twisting of pores are caused by the buckling and bending deformation of porous walls, and depend on the orientation of loading (Supplementary Figure S13).

**Figure 4:**
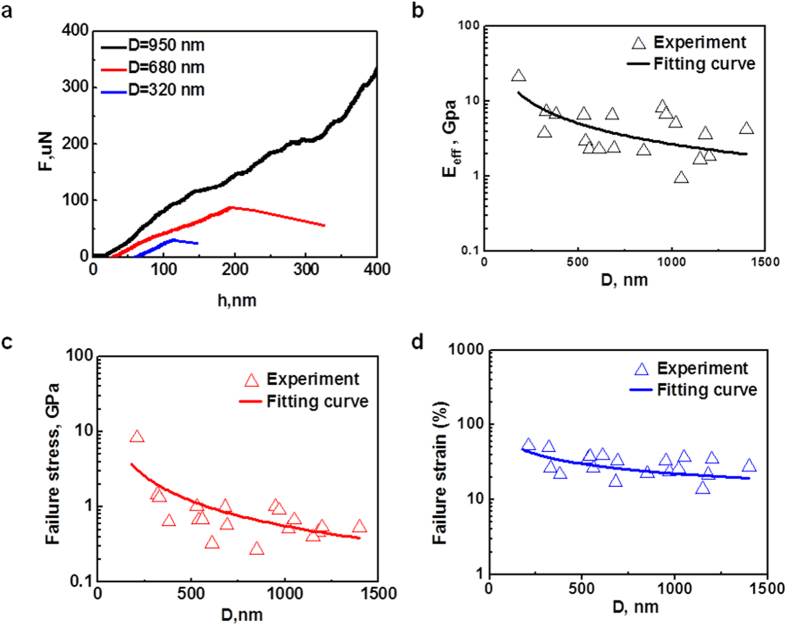
Size effect of porous carbon nanospheres on their corresponding mechanical properties. (**a**) Compressive force and displacement curves for mesoporous carbon spheres with three representative diameters (*D* = 950 nm, 680 nm, and 320 nm), and variation of the measured (**b**) Young’s modulus, (**c**) failure stress, and (**d**) failure strain with different sizes from *in situ* compression tests.

The compression experiments on porous carbon spheres with different diameters were also performed to investigate the effect of size on the mechanical properties, and amorphous carbon nanospheres with similar densities of interior pores were employed. The force and displacement curves of mesoporous carbon nanospheres are shown in Fig. 4a. An approximately linear-increase of force with displacement followed by a sudden drop due to the failure of the entire structure, is observed in a smaller carbon nanosphere (*D* = 320 nm). As the size of the carbon sphere increases to 950 nm, a force plateau appears after the initial elastic deformation and extends all the way until the densification of collapsed pores occurs with a steep rise of the compressive force. Generally, smaller spheres are more like solid amorphous carbons, and bending, buckling and crushing of pores may be weakened and a failure similar to that of solid carbon spheres is expected with a sudden drop of force. The similar size effect has also been confirmed in our FEA (Supplementary Figure S14). To further elucidate this unprecedented size effect, we define the force and displacement at the transition point from buckling and collapse to densification stage of pores as failure strength, 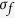, and compressive strain, 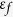, respectively. They are defined by


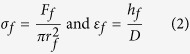


where 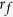 and 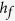
are the contact radius of nanosphere to the flat indenter and applied compressive displacement at the failure load, 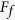, respectively. Figure 4b–d shows that smaller spheres have higher measured Young’s moduli, 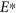, higher failure strengths, 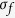, and larger failure strains, 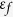. Particularly, as indicated in Fig. 4d, the maximum failure strain under all current considerations (sphere size, *D* = 210 nm) can be as high as 52%, and the corresponding failure stress of 8.3 GPa, which is 10 times higher than that of solid graphite ( < 4.5% in failure strain, and < 350 MPa in failure stress)^30^,^31^,^32^. According to the classic Gibson and Ashby’s model^32^, 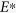 is a function of relative density of the porous sphere, 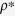 and solid sphere, 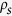 via


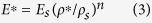


where 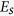 is the intrinsic Young’s modulus of solid carbon, 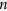 is a scaling exponent. Meanwhile, as shown in Figure S15 in the supplementary information, 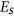 increases with decreasing solid particle size. Similar with other nanoparticles such as polystyrene nanospheres at the nanoscale^33^, this strong scaling effect can be approximately described via^34^


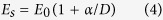


where 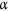
is the scaling constant, and 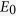 is Young’s modulus of solid carbon in macro scale. Thus, the classic Gibson and Ashby’s model becomes


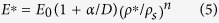


Considering the mesoporous sphere with density 0.26 g/cm^3^ and solid sphere with averaged density 2.2 g/cm^3^, the strong size dependent 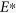 is well predicated. Besides, the scaling exponent 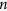
is 0.82
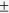
0.2 and smaller than that of uniform porous structures (1 ~ 3)^35^,^36^, consistent with distribution of disordered open and closed nanopores and interaction between shell and pores in spheres. Similarly, the size effect of failure strain and stress in porous carbon spheres (Fig. 4c,d) is also believed to result from the size dependence of failure strain and stress in their solid carbon nanosphere counterpart. In addition to the contribution of solid sphere at the nanoscale to the scaling effect of porous spheres, the thickness of outer shell in porous spheres is also expected to be critical. For instance, with the variation of sphere diameter-to-shell thickness ratios, the failure of spheres may transit from fracture flexure to buckling^37^. Besides, the present porous structures consist of both open- and closed- pores with no-uniform distributions, and there is a thin cell wall in mescoporous spheres (Supplementary Figure S2). At the nanoscale, the thin cell wall usually has an enhanced strength and may also fail without bending or buckling deformation^38^. A further accurate scaling model with incorporation of such as pore distribution, shape and wall size will be expected by optimizing the porous synthesis processing with controllable porous structures.

**Figure 5:**
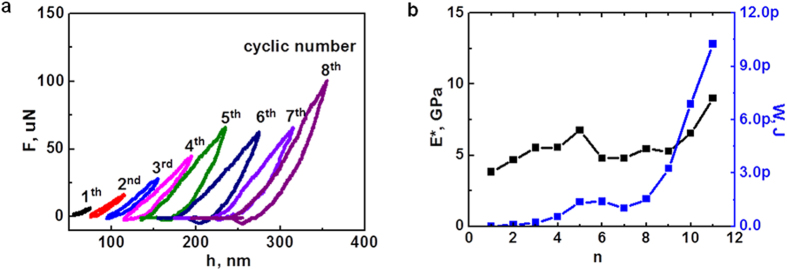
Mechanical behaviors of carbon nanospheres under a cyclic displacement loading during compression tests. (**a**) Force and displacement curves at selected loading cycles, where a curve shift of 20 nm from previous one is made for clear presentation, and (**b**) variation of the measured Young’s modulus and energy dissipation under different cyclic loadings.

A cyclic compression experiment on a mesoporous carbon nanosphere (*D* = 950 nm) was further performed to estimate the energy dissipation at different porous deformation (Supplementary Video 7). The maximum compression in each loading cycle increased by 20 nm up to a total of 220 nm, and both loading and unloading rates were kept at 1 nm/s. Figure 5a shows the force-displacement curve obtained by compression on the mesoporous sphere under selected cyclic numbers (a complete force-displacement curve is provided in Supplementary Figure S16). At a small displacement, a continuous and completely closed curve is observed, indicating a complete recovery of deformation after unloading. When the maximum compression reaches 100 nm (fifth cycle), the unloading curve cannot rebound to the initial point, indicating irreversible buckling and the collapse of inner pores starts to occur. In the sixth and seventh cycle, the maximum force remains almost the same, which is in good agreement with the deformation mechanism of bending, buckling, twisting and collapse of most inner pores. Finally, at the eighth cycle, the maximum force shows an obvious increase due to the densification of the collapsed pores, also echoing well with above deformation behavior at monotonic loadings. Similar to most porous or cellular materials, a hysteresis loop is found in each loading-unloading cycle, implying an obvious energy dissipation due to the deformation of pores. The measured Young’s modulus, 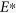, and energy dissipation, *W*, in each cycle are extracted and plotted in Fig. 5b (Supplementary Note 3). An increased energy dissipation with increasing cyclic number is obtained due to the deformation of more pores at each cycle. As more pores deform and collapse, the porous sphere will become dense, leading to an increase of 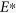. In particular, the rapid increase of both 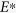 and *W* beyond the deformation plateau (the eighth cycle) agrees with a quick densification of pores. Since the loading manner is quasistatic in each cycle at the room temperature and the applied averaged stress is very low, it is expected that the compression will not cause significant changes to the amorphous structure of carbon nanospheres.

## Discussion

In summary, we demonstrate that inherently brittle amorphous carbon materials can simultaneously exhibit high strength and large compressive deformation with well-defined nanostructures. This is achieved through a bioinspired hybrid design of zero-dimensional porous carbon spheres with nacre-like outer shell morphology and honeycomb-like inner nanoporous structure. Porous carbon nanospheres with amorphous structures are capable of deforming through bending, buckling, twisting, rotation and collapse of pores improving the energy dissipation/absorption capacity relative to solid or hollow carbon spheres. Both experimental and theoretical analyses indicate that hierarchical architectures and microstructures of materials have critical connections with their mechanical properties. It is envisioned that such porous nanospheres with low-cost and high throughput productions will serve as elementary building blocks of assembling advanced structures and unlock a vast array of potential applications capable of meeting emerging needs in next-generation wearable electronics, sustainable electrodes, flexible supercapacitors, multifunctional filters, smart sensors and actuators requiring enhanced deformation and/or strength.

## Methods

### Synthesis and characterization of porous carbon spheres

The experimental setup used in this work to synthesize amorphous carbon nanospheres (Fig. 1) is shown in Figure S1. Argon was used as the carrier gas at a flow rate of 1 L/min. The carrier gas was flowed through the reactor for at least 30 min to purge the system prior to the addition of the precursor solution which was prepared by stoichiometrically mixing propiolic acid (95% from sigma-aldrich) with coressponding alkali hydroxides (99.99% from sigma-aldrich). The obtained black powders were washed and centrifuged with deionized water for at least 5 times to remove any salt formed during synthesis. The N_2_ adsorption-desorption was carried out using Micromeritics ASAP 2020 physisorption analyzer to determine the density of porous carbon nanospehres studied in this work. TEM images were taken with a JEOL 2100 transmission electron microscope with an accelerating voltage of 200 kV. SEM images were taken using a Hitachi S4800 field-emission scanning electron microscope with an accelerating voltage of 10 kV. Powder X-ray diffraction patterns (PXRD) of the product were obtained with a Japan Rigaku DMax-γA rotation anode X-ray diffractometer equipped with graphite monochromatized Cu Kα radiation (λ = 1.54178 Å). Raman spectra were obtained directly from a thin film of carbon samples deposited on Si wafers on a Horiba Jobin-Yvon LabRAM Raman Spectrometer (excited with a 532 nm laser).

### *In situ* compression experiment

The porous carbon spheres were initially dispersed in ethanol and the suspensions were sonicated for 30 seconds to obtain a homogeneous solution. A silicon wedge substrate with 1 μm wide top surface (Hysitron, Inc) was dipped coated by the prepared solution, and then dried under an infrared heating lamp for 2 mins to minimize “necking” at contact between wedge and sphere. The prepared silicon wedge was first checked in the SEM to see if the nanosphere was well positioned for contact without obvious “necking” between them. The wedge was mounted on copper holder (Hysitron, Inc) with crystal bond to ensure strong adhesion as well as to ensure the wedge surface was suitably parallel with electron beam under TEM. The holder was then mounted on picoindenter to achieve a good alignment between flat punch and wedge.

The *in situ* TEM compression experiments were carried out using a Hysitron PI-95 picoindenter (Hysitron, Inc) in a JOEL 2010 LaB6. The displacement control mode was used for compression on all carbon spheres. A conductive diamond flat punch with 3.0 μm in diameter (Micro Star Tech, Inc) was used. In order to record the entire deformation process (bending, buckling, twisting, rotating, and collapsing) of each individual single carbon nanosphere, a loading speed of 1 nm/s which is the mimiumum loading tolerence of the system that can be considered as a quasistatic loading was employed during the experiment. Typical air indentations were carried out to calibrate the transducer before each experiment.

### Finite element analysis

Finite element analyses (FEA) were performed using ABAQUS to reveal deformation mechanisms and capture key mechanical parameters. A full 3D nanosphere model was employed and verification of mesh density was carefully checked for each model size. Both flat punch and substrate were assumed to be rigid, and mechanical behavior of amorphous carbon materials are described by elastic model with Young’s modulus of 20.2 GPa and Poisson’s ratio of 0.25^39^, where Young’s modulus is obtained from compression experiment on solid nanosphere through Hertz contact theory (Supplementary Figs S6 and S7, Supplementary Note 1). Quasistatic displacement loading was applied, and a typical Coulomb friction coefficient at contacts among flat punch, sphere and substrate was taken to be 0.02 for contacts.

## Additional Information

**How to cite this article**: Yang, W. *et al.* Large-deformation and high-strength amorphous porous carbon nanospheres. *Sci. Rep.* **6**, 24187; doi: 10.1038/srep24187 (2016).

## Supplementary Material

Supplementary Information

Supplementary Movie 1

Supplementary Movie 2

Supplementary Movie 3

Supplementary Movie 4

Supplementary Movie 5

Supplementary Movie 6

Supplementary Movie 7
