# Supplementary material for: Large-deformation and high-strength amorphous porous carbon nanospheres
Source: Sci Rep. 2016 Apr 13;6:24187. doi: 10.1038/srep24187 (PMC4829827; doi:10.1038/srep24187)
Supplement: Supplementary Information [file srep24187-s1.pdf]

Supporting Information

Large-deformation and high-strength amorphous porous carbon nanospheres

Weizhu Yang[1†], Shimin Mao[2†], Jia Yang[3†], Tao Shang[2], Hongguang Song[3], James Mabon[2], Wacek Swiech[2], John R Vance[2], Zhufeng Yue[4], Shen J. Dillon[2], Hangxun Xu[3*], Baoxing Xu[1,5*]*

[1]Department of Mechanical and Aerospace Engineering, University of Virginia, Charlottesville, VA 22904, USA.

[2]Departments of Materials Science and Engineering, University of Illinois at Urbana-Champaign, Urbana, IL 61801, USA.

[3]CAS Key Laboratory of Soft Matter Chemistry, Department of Polymer Science and Engineering, University of Science and Technology of China, Hefei, Anhui 230026, China.

[4]Department of Engineering Mechanics, Northwestern Polytechnical University, Xi'an, Shannxi 710072, China.

[5]Institute for Nanoscale and Quantum Scientific and Technological Advanced Research, University of Virginia, Charlottesville, VA 22904, USA.

†These authors contributed equally to this work.

* *Correspondence to S. D. (sdillon@illinois.edu), H. X. (hxu@ustc.edu.cn) or B. X. (*bx4c@virginia.edu*)*

Note 1: Determination of Young's modulus of solid carbon nanosphere

Under *in situ* compression experiments, the diamond flat indenter is assumed to be rigid. **Figure S6** shows the schematic of a deformable solid hemisphere upon compression. The diameter of solid sphere is D and after the displacement of compression, h, the contact radius of flat indenter with deformed surface is a. According to the classic Hertz contact theory, the relationship between compression depth, $h/2$, and force, F, is

$$F = \frac{4}{3}\frac{E}{1-\nu^2}\left(\frac{D}{2}\right)^{1/2}\left(\frac{h}{2}\right)^{3/2} \tag{S1}$$

Where ν is the Poisson's ratio and is taken 0.25 for amorphous carbons.

Figure S7 shows the force and displacement curve of compression on a solid carbon sphere with diameter of 800nm, and the compression from 50nm to 200nm was used to fit the experimental curve using **Equation S1**. The Young's modulus of $E = 20.2 \pm 3.0$ GPa was extracted, and used in all FE simulations. An excellent agreement between FEA and experiment on the solid carbon sphere is obtained in **Figure S7**, and also observed in compression on hollow spheres in **Figure S9**, verifying both experiments and FE models.

Note 2: Finite element analysis (FEA)

Finite element analysis (FEA) was performed using ABAQUS. Full 3D nanosphere model was employed in FEA and verification of mesh density was carefully checked in analysis for each model size. Both of the flat punch and substrate were assumed to be rigid, and the mechanical behavior of amorphous carbon is described by an elastic model with Young's modulus of 20.2 GPa and Poisson's ratio of 0.25, where Young's modulus is obtained from the compression experiment performed on a solid carbon nanosphere through Hertz contact theory (**Note 1**). A quasistatic displacement loading was applied, and the Coulomb friction coefficient was taken to be 0.02 at contacts among flat punch, sphere and substrate.

When the number of inner nanopores is more than 1, all nanopores were placed symmetrically inside the spheres. **Figure S8** shows the FE models and schematics of pores inside, where each red point represents the central position of nanopore. Their central positions did not change with their size. In the simulation, nanospheres with 125 nanopores and 21 nanopores were used to model the mesoporous and macroporous nanosphere, respectively. The loading direction vertical to the connection edge of two neighboring nanopores (**Figure S13**) was used unless a specific definition.

Note 3: Determination of the measured Young's modulus of porous nanosphere

Equation S1 is employed to extract the Young's modulus of porous nanospheres at a small displacement range (<100nm), and it reflects the total stiffness of nanoporous spherical structures, referred to as the measured Young's modulus, E^*, in the present study. The same method is also employed to extract the measured Young's modulus in the cyclic compression experiment. As for comparisons, the unloading curves are also employed to extract the measured Young's modulus through Oliver and Pharr's indentation method, and they show a good consistence with extractions from **Equation S1** (**Figure S16b**)

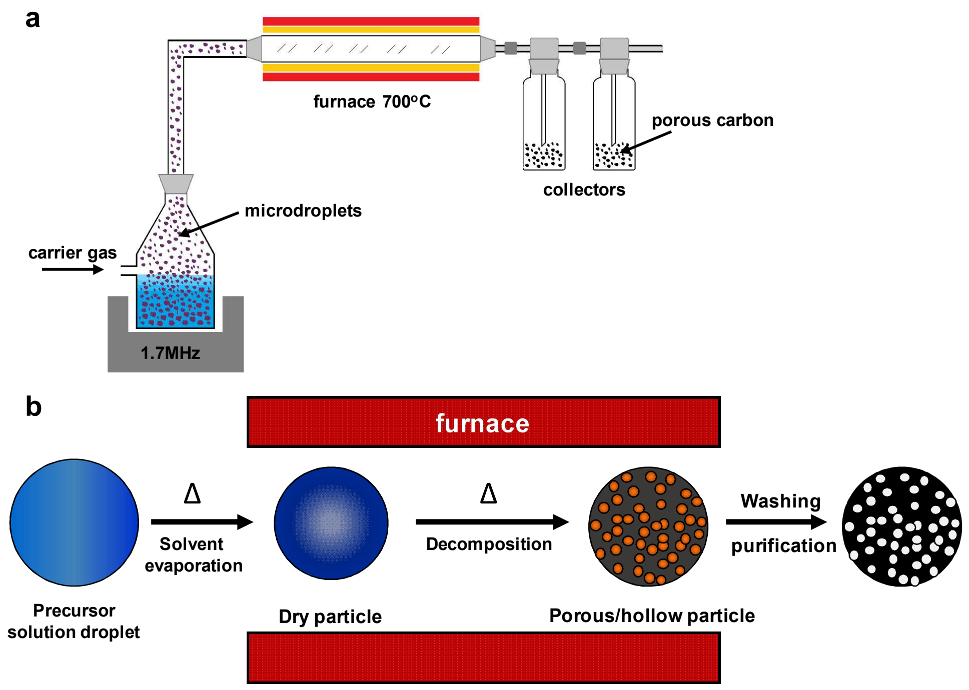

Figure S1 (a) Schematic illustration of the ultrasonic spray pyrolysis setup used for synthesizing porous carbon nanospheres studied in this work. (b) Simplified schematic illustration of the formation process for porous/hollow carbon spheres.

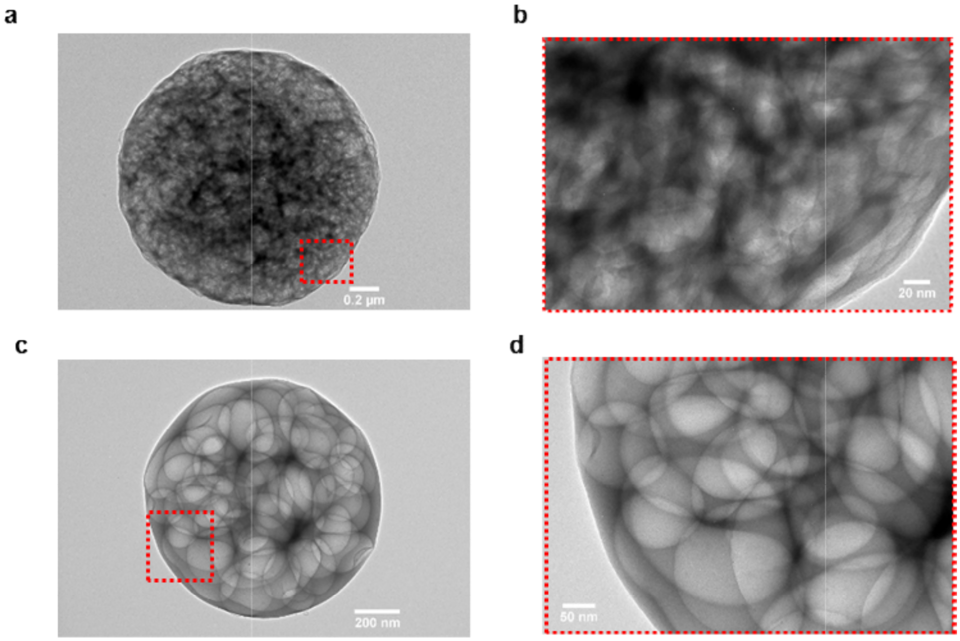

Figure S2 TEM images of mesoporous (a, b) and macroporous (c, d) carbon nanospheres studied in this work.

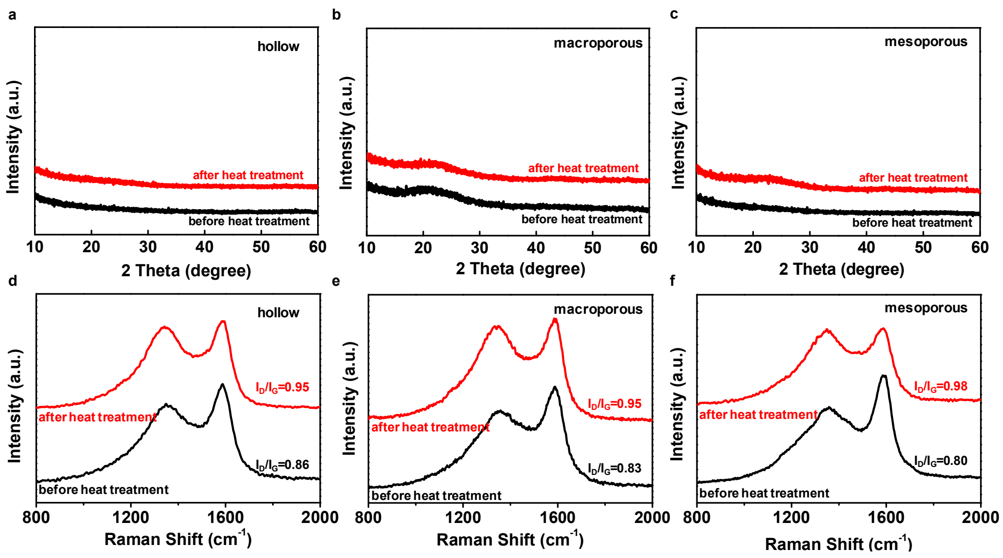

Figure S3 (a-c) Powder X-ray diffraction patterns (PXRD) and (d-f) Raman spectra of porous carbon nanospheres (before and after heat treatment) with different structures. PXRD indicates carbon nanospheres remains amorphous after heat treatment.

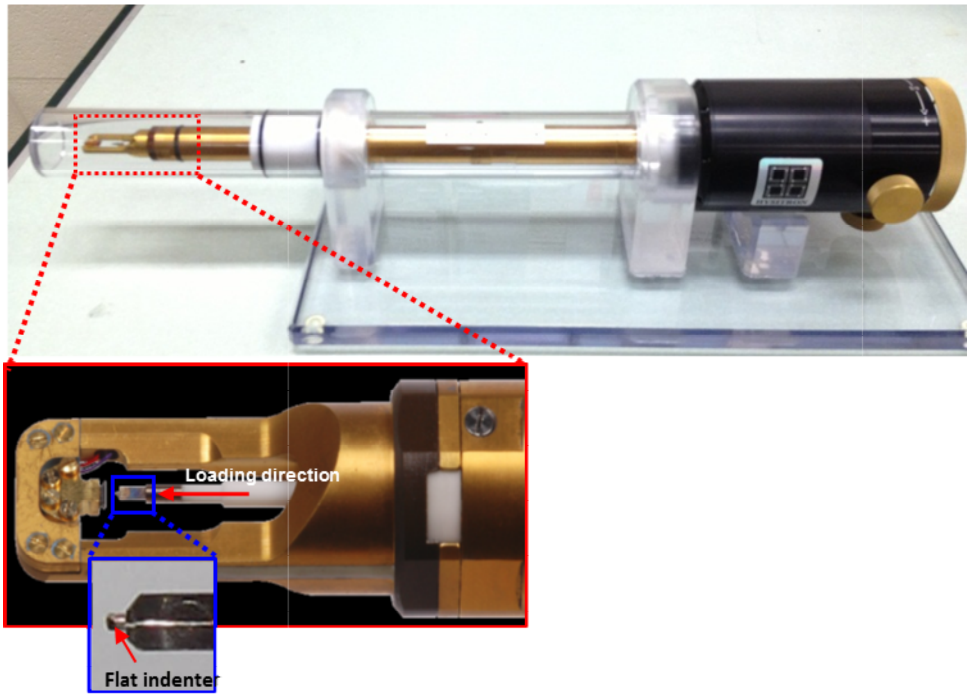

Figure S4 Photograph of a Hysitron PI-95 TEM holder used in this work.

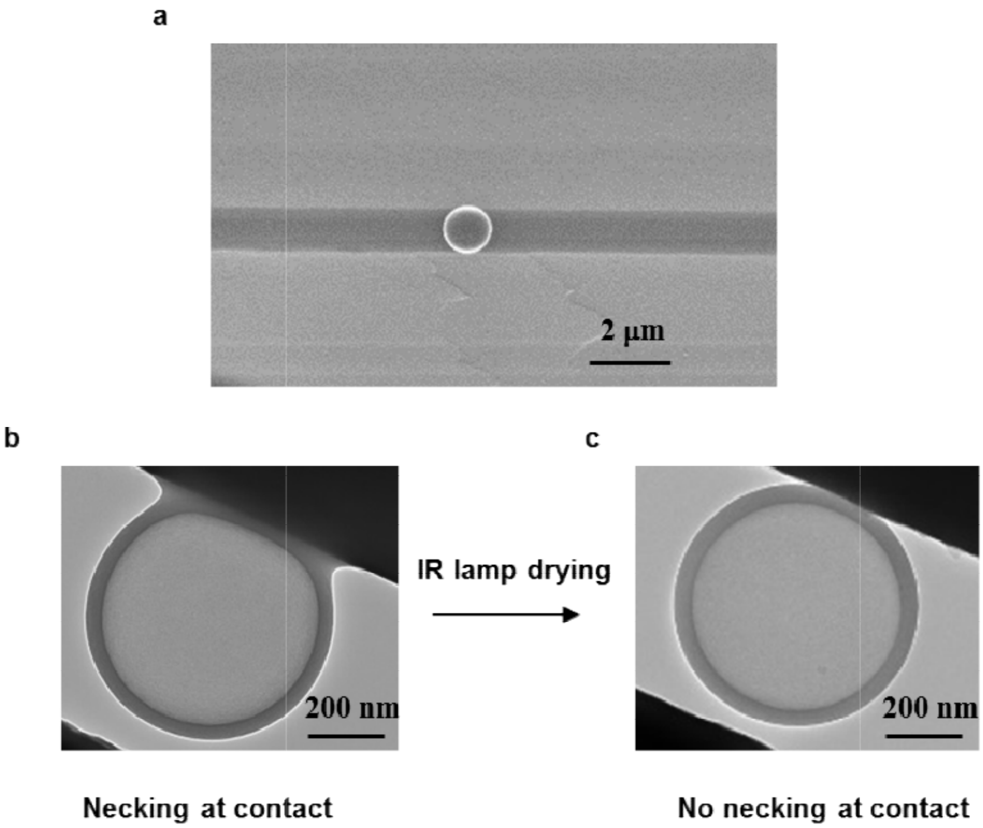

Figure S5 (a) SEM image of a carbon nanosphere sitting at the middle of the silicon wedge substrate. (b) A necking phenomenon is observed at contact point which can be minimized through IR lamp drying.

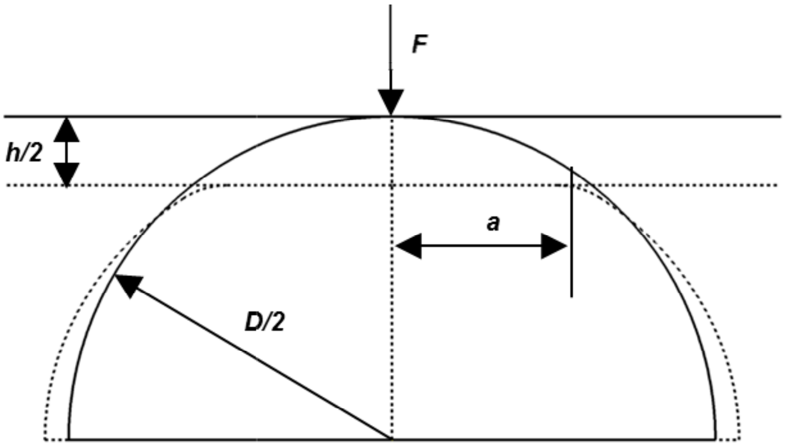

Figure S6 Schematic of a deformable hemisphere before (solid curve) and after (dash curve) pressed by a rigid and frictionless flat punch. The original diameter of sphere is *D*. The indentation displacement is *h*/2, and contact radius is *a*, corresponding to a contact force *F*.

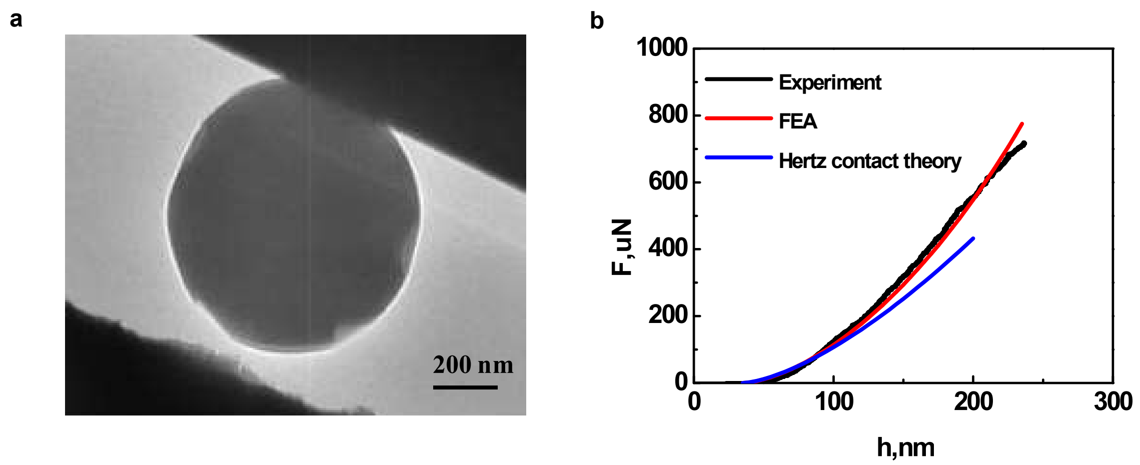

Figure S7 (a) TEM image of a carbon sphere positioned on a silicon wedge substrate before compression. (b) Force and displacement curves from experiment, Hertz contact theory and FE predication, where Young's modulus of amorphous carbon materials is extracted based on Hertz contact theory and is used in FEA to generate the FE predication curve.

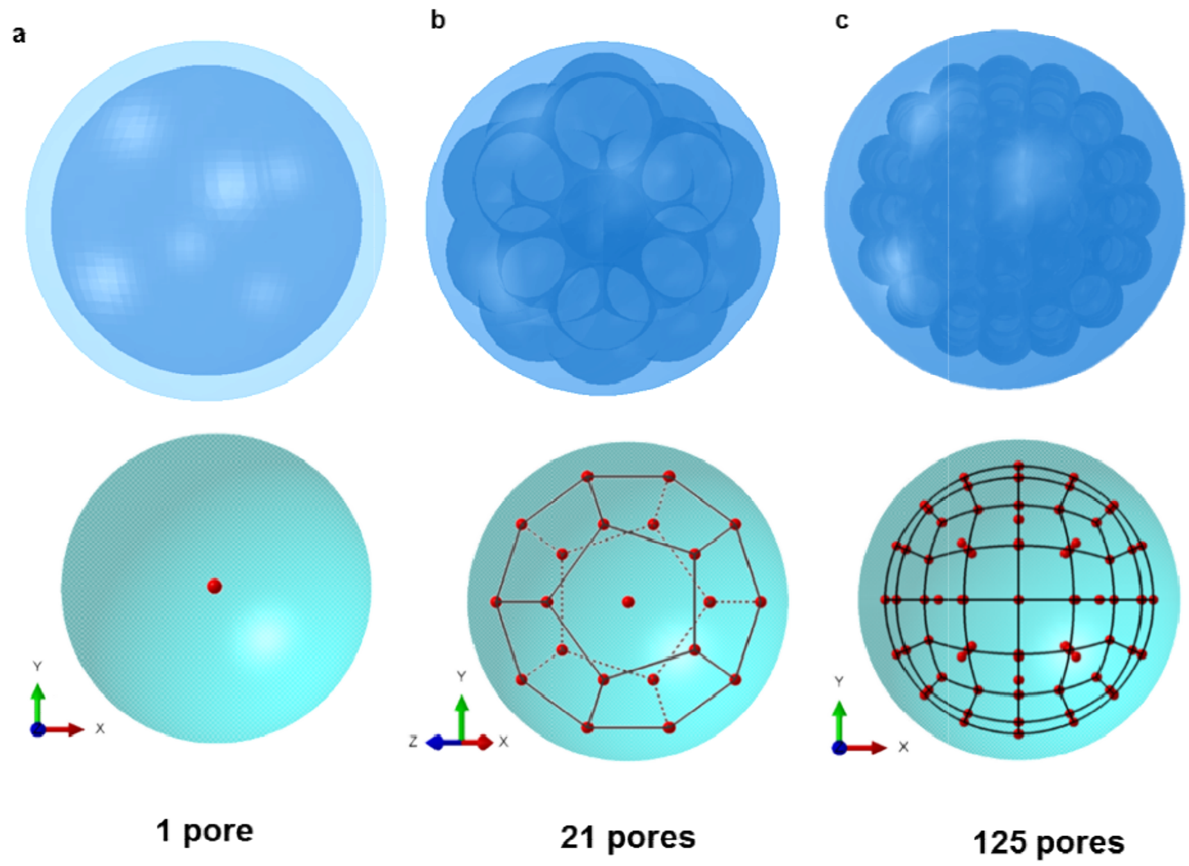

Figure S8 (a) A hollow sphere for modeling the synthesized hollow carbon nanosphere, (b) a sphere with 21 pores symmetrically positioned inside to represent the synthesized macroporous carbon nanosphere, and (c) a sphere with 125 pores symmetrically positioned inside to represent the synthesized mesoporous carbon nanosphere. The red points indicate the central position of nanopores inside each sphere.

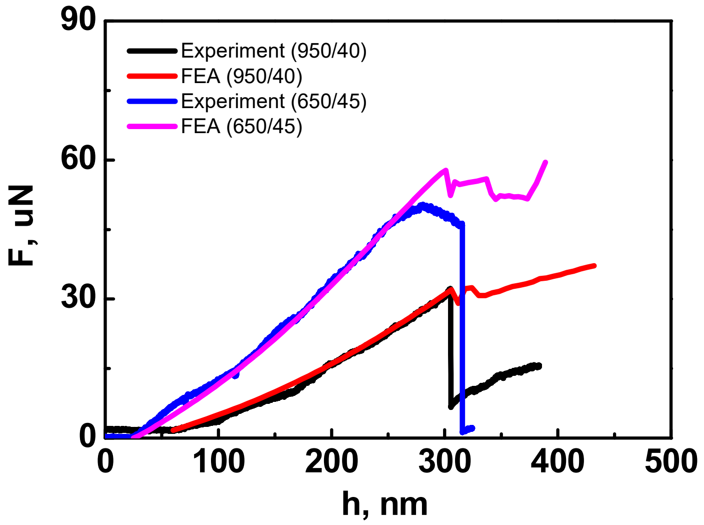

Figure S9 Comparison of force and displacement curves between experiments and FEA on hollow carbon spheres with different diameters/shell thicknesses.

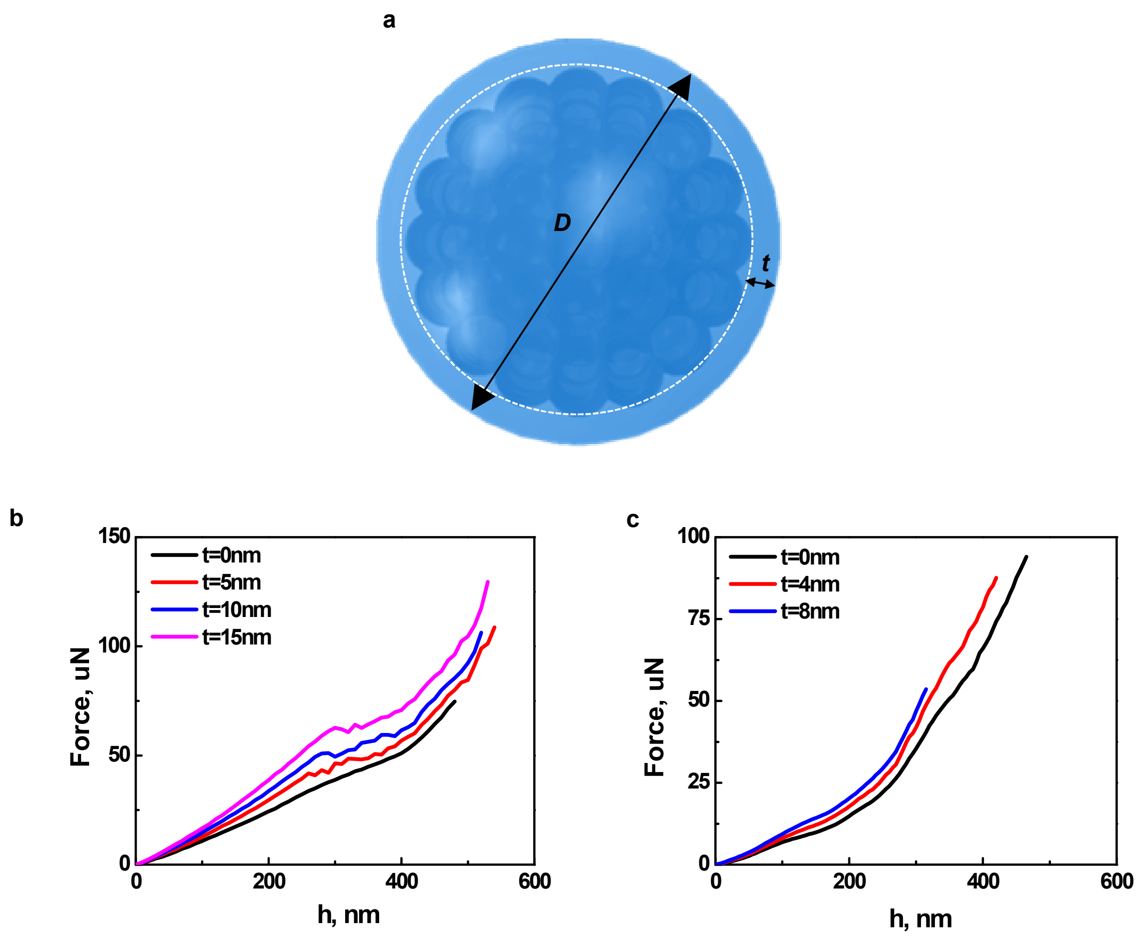

Figure S10 (a) Schematic illustration of porous carbon nanosphere, where *D* is the diameter of nanosphere, and *t* is the outer shell thickness. (b) Compressive force-displacement curves of macroporous carbon nanospheres (*D* = 950 nm) with different outer shell thicknesses (*t*), where macroporous nanospheres consist of 21 inner nanopores in FE simulations. (c) Compressive force-displacement curves of mesoporous carbon nanospheres (*D* = 950 nm) with different outer shell thicknesses (*t*), where mesoporous nanospheres consist of 125 inner nanopores in FE simulations.

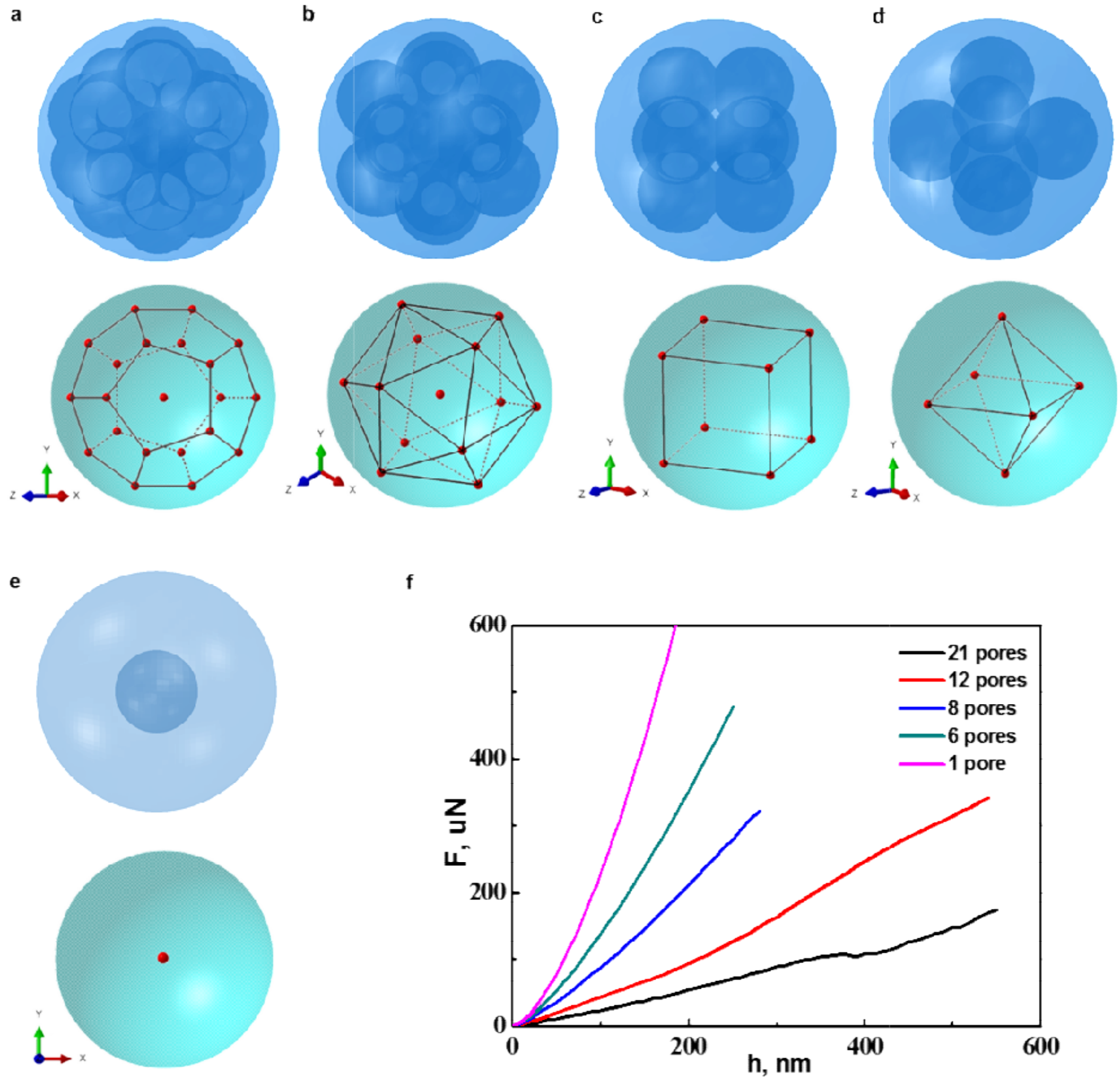

Figure S11 (a)-(e) FEA models of carbon nanospheres (950 nm in diameter) with different numbers of pores (from 21 to 1). The red point in each sphere indicates the central position of a nanopore. (f) Force and displacement curves from compression on carbon nanospheres with different numbers of pores inside.

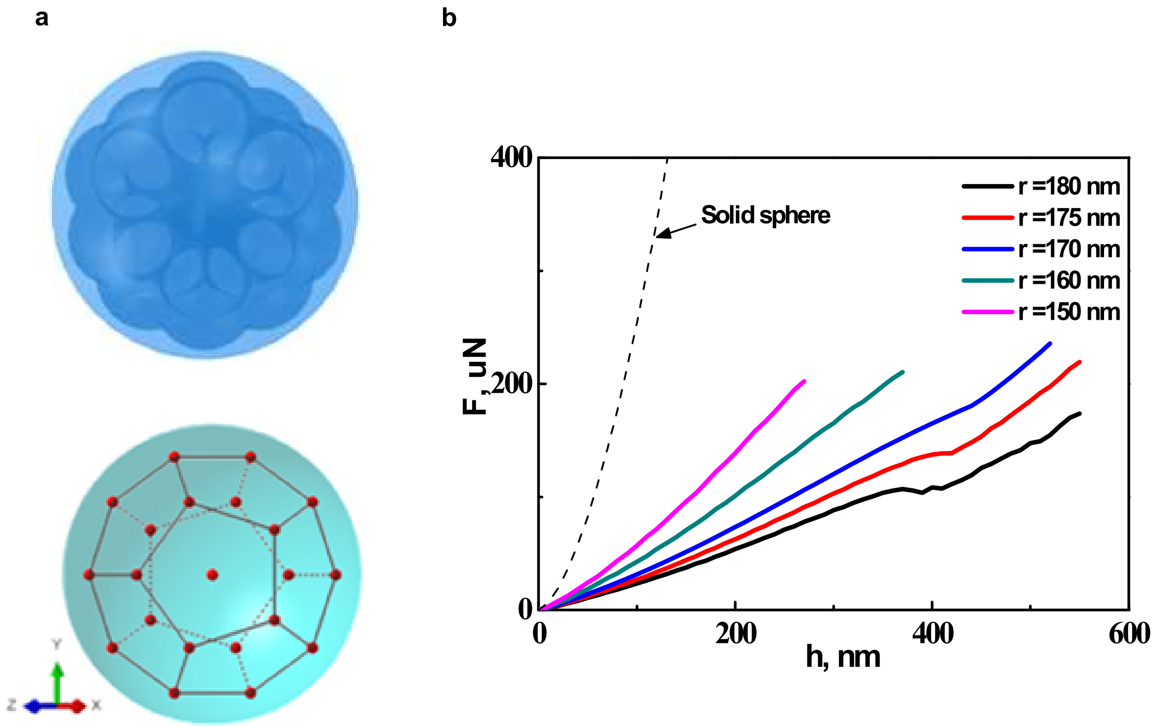

Figure S12 (a) FEM model of a carbon nanosphere (950 nm in diameter) embedded with 21 nanopores, and (b) relationship of force and displacement from compression on a carbon nanosphere with the same number of nanopores but different pore radii (*r*), where the dash curve shows the result from a solid sphere.

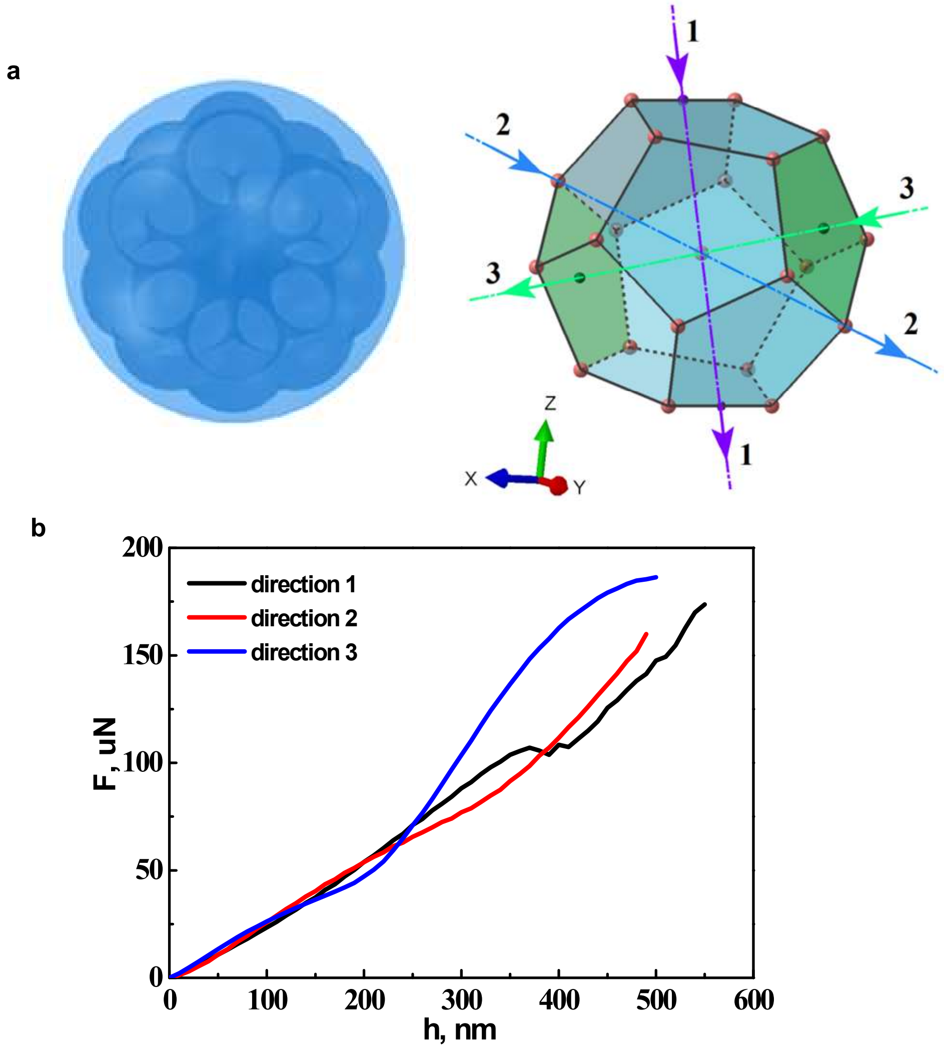

Figure S13 (a) FEA model of a carbon nanosphere (950 nm) with 21 nanopores, (b) illustration of three displacement loading directions used in FEA (the red points represent the central positions of nanopores), and (c) force and displacement responses at three different loading directions.

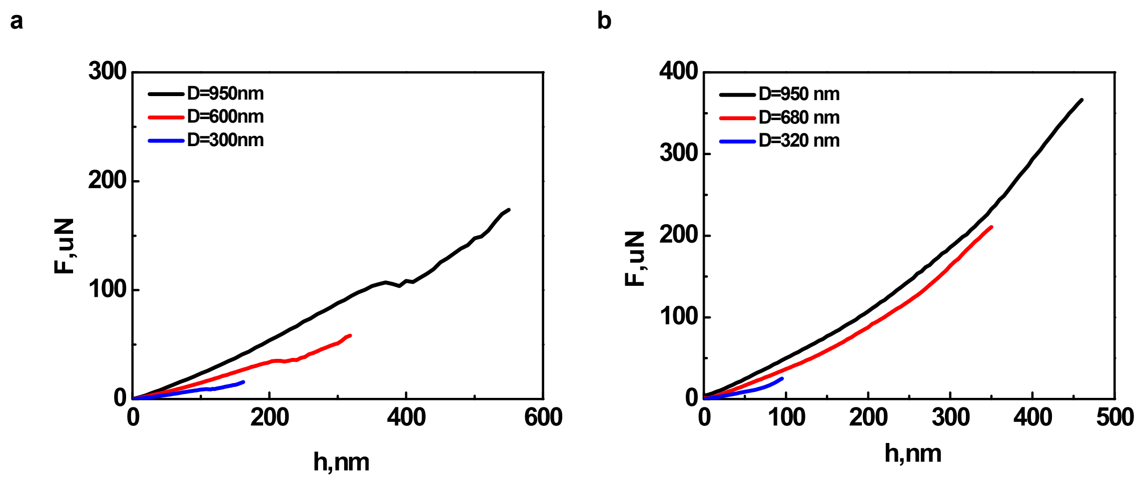

Figure S14 (a) The displacement and force curves from FEA on carbon nanospheres with different diameters (*D*), where 21 nanopores are used to represent the macroporous structure. (b) The displacement and force curves from FEA on carbon nanospheres with different diameters (*D*), where 125 nanopores are used to represent the mesoporous structure.

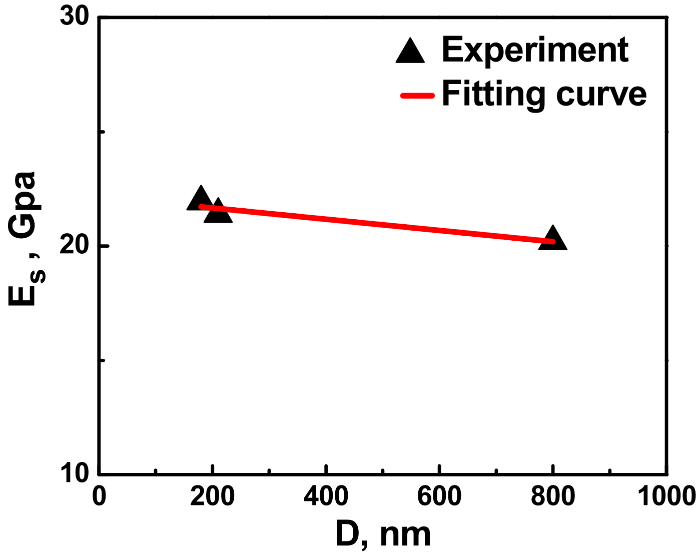

Figure S15 Variation of Young's modulus of solid carbon nanospheres with their diameters.

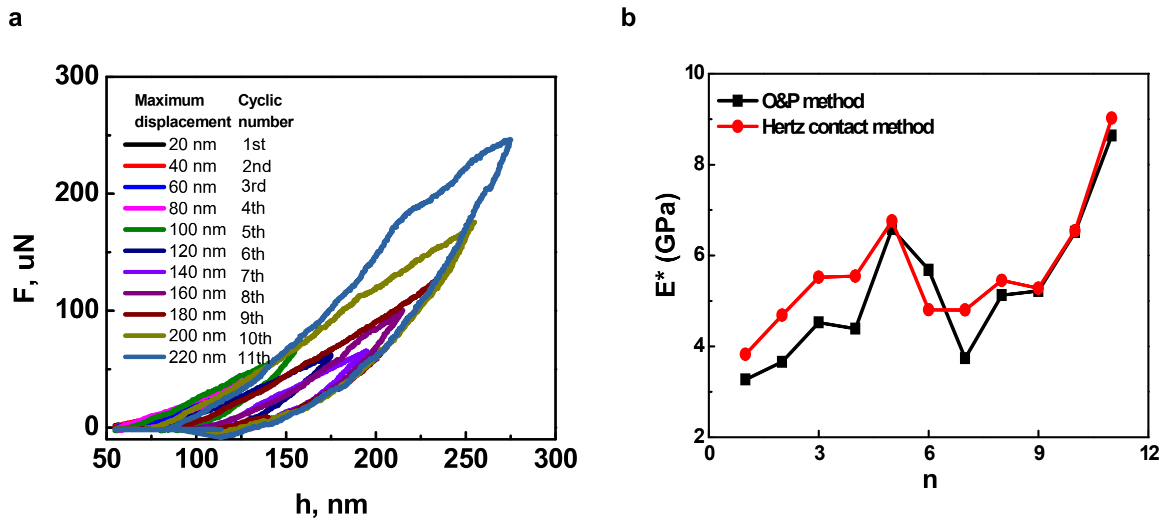

Figure S16 (a) Force-displacement curves of a 950 nm carbon nanosphere under the cyclic loading condition. (b) Variation of measured Young's modulus, E^*, with cyclic numbers through both Oliver-Pharr's method on the basis of unloading part (back curve) and Hertz contact theory on the basis of loading part (red curve).

Captions of Supplementary Videos

Video 1: Movie of the compression experiment on a macroporous carbon nanosphere. The original frame rate is 1 frame per second and the frame rate is increased by 10X here.

Video 2: Movie of the **c**ompression experiment on a hollow carbon nanosphere. The original frame rate is 1 frame per second and the frame rate is increased by 10X here.

Video 3: Movie of the compression experiment on a mesoporous carbon nanosphere. The original frame rate is 1 frame per second and the frame rate is increased by 10X here.

Video 4: Movie of the finite element analysis of compression on a hollow carbon nanosphere.

Video 5: Movie of the finite element analysis of compression on a carbon nanosphere with 21 pores inside.

Video 6: Movie of the finite element analysis of compression on a carbon nanosphere with 125 pores inside.

Video 7: Movie of the cyclic compression experiment on a mesoporous carbon nanosphere. The original frame rate is 1 frame per second and the frame rate is increased by 10X here.
